# Predictive Value of a Radiomics-Derived Risk Score for Local Progression in T3 Laryngeal Cancer: A 10-Year Single-Center Retrospective Cohort Study

**DOI:** 10.3390/jcm15041511

**Published:** 2026-02-14

**Authors:** Caglar Eker, Muhammed Dagkiran, Emin Demirel, Burak Mete, Hasan Suat Arslantas, Omer Kaya, Bedir Kaya, Elvan Onan, Naqibullah Mohammadi, Mustafa Mert Gedik, Ilda Tanrisever Pehlivan, Merve Gizem Gonullu, Ozgur Surmelioglu

**Affiliations:** 1 Department of Otolaryngology and Head and Neck Surgery, Faculty of Medicine, Cukurova University, Adana 01330, Turkey; muhammeddagkiran@gmail.com (M.D.); uygurelvan@hotmail.com (E.O.); nakibmohammadi01@gmail.com (N.M.); mrtgdk01@gmail.com (M.M.G.); ildatanrisever@gmail.com (I.T.P.); surmeli2004@yahoo.com (O.S.); 2 Department of Radiology, Faculty of Medicine, Afyonkarahisar University of Health Sciences, Afyon 03030, Turkey; dremindemirel@gmail.com; 3 Department of Public Health, Faculty of Medicine, Cukurova University, Adana 01330, Turkey; bmete@cu.edu.tr; 4 Department of Radiation Oncology, Faculty of Medicine, Cukurova University, Adana 01330, Turkey; hsuatarslantas@yahoo.com (H.S.A.); mervegizem1@gmail.com (M.G.G.); 5 Department of Radiology, Faculty of Medicine, Cukurova University, Adana 01330, Turkey; dr.omerkaya@gmail.com (O.K.); bedirkaya00@gmail.com (B.K.)

**Keywords:** T3 laryngeal cancer, radiomics, local progression, chemoradiotherapy, texture analysis

## Abstract

**Background/Objective:** Local progression after concurrent chemoradiotherapy in T3 laryngeal carcinoma (LC) remains difficult to predict using conventional clinical assessment alone. This study aimed to develop a radiomics-derived risk score from routine post-treatment contrast-enhanced CT and evaluate its prognostic value—together with clinical variables—for predicting local progression-free survival (LPFS). **Methods:** In this single-center retrospective cohort, 67 patients with pathologically confirmed T3-stage LC treated with chemoradiotherapy were included. All patients underwent contrast-enhanced CT at baseline and 3 months after treatment completion; radiomics analysis was performed using post-treatment CT with 3D manual segmentation of the primary tumor. A total of 111 radiomic features were extracted (shape, first-order, and texture). Features with AUC > 0.60 were screened, and six top-performing features were used to construct a radiomics score (0–6) based on optimized cutoffs. The primary endpoint was LPFS, defined as time from end of treatment to biopsy-proven residual or recurrent primary tumor. Cox regression and Kaplan–Meier analyses were performed. **Results:** Mean age was 59.6 ± 9.4 years, and 37.3% developed local progression during follow-up. In multivariable Cox analysis, the radiomics score remained an independent predictor of local progression (HR per 1-point increase: 2.38; 95% CI: 1.59–3.56; *p* < 0.001), with high model discrimination (C-index: 0.855). LPFS differed significantly across radiomics score strata (*p* < 0.001); higher scores were associated with substantially shorter time to progression and poorer 1-, 3-, and 5-year LPFS rates. **Conclusions:** A post-treatment CT-derived radiomics score integrated with clinical parameters showed favorable performance for predicting local progression in T3 laryngeal cancer after chemoradiotherapy. Although external validation is required, this approach may support more individualized surveillance by identifying patients at higher risk of early treatment failure.

## 1. Introduction

Notwithstanding a recent decline in laryngeal cancer (LC) incidence, it remains a significant healthcare burden [1]. According to the WHO/IARC Global Cancer Observatory (GLOBOCAN 2022), laryngeal cancer accounted for 189,191 new cases and 103,359 deaths worldwide in 2022, underscoring its ongoing global impact [2]. Moreover, demographic projections indicate that the overall global cancer burden is expected to increase substantially by 2050, reinforcing the need for improved risk stratification and optimized post-treatment surveillance strategies [3].

While early-stage tumors can be managed effectively with transoral surgery or radiotherapy, advanced-stage LC often necessitates multimodal approaches [4,5]. The goal in such cases is to eradicate disease while maximizing laryngeal function preservation. Landmark studies by the Veterans Affairs and the Radiation Therapy Oncology Group contributed to a paradigm shift toward non-surgical organ preservation strategies for advanced LC, aiming to avoid the morbidity of total laryngectomy [6,7]. However, despite these advancements, overall survival rates for LC have paradoxically declined over the past decades [8]. Evidence suggests that locoregional recurrence following organ preservation strategies remains high, with 5-year rates nearing 30–40% [9,10,11]. Some studies have linked this to the limitations of non-surgical approaches in certain tumor subtypes [8,12].

Laryngeal cancer staging is largely determined by vocal fold mobility and the anatomic extent of tumor spread. Within the American Joint Committee on Cancer (AJCC) TNM system, advanced-stage LC is traditionally defined by the presence of T3–T4 disease, regardless of lymph node status [13]. Among these, T3 tumors constitute a particularly heterogeneous category, encompassing lesions with vocal fold fixation and/or invasion of the paraglottic or pre-epiglottic spaces, with variable patterns of cartilage involvement.

Although TNM staging remains the most commonly used prognostic framework for long-term outcomes, its reliance on clinicopathological descriptors alone limits its ability to capture biologic aggressiveness and to predict treatment response, particularly after organ-preservation strategies such as chemoradiotherapy (CRT) [12,14]. Accordingly, there has been increasing interest in the development of multivariable predictive models that integrate clinical features with molecular biomarkers and quantitative imaging-derived radiomic signatures to improve individualized risk stratification. However, existing evidence is largely derived from heterogeneous head and neck cancer (HNC) cohorts without adequate site-specific stratification, while larynx-focused studies have often evaluated mixed-stage populations, limiting the generalizability of proposed models to clinically relevant subgroups such as T3 disease [15].

Another challenge following CRT is the treatment-induced tissue alterations, including ischemia, edema, inflammation, and delayed fibrosis [16]. These changes complicate post-treatment evaluation and hinder the differentiation between radiation-related effects and tumor progression. In some cases, repeated biopsies may remain inconclusive, potentially leading to delays in diagnosis and timely intervention.

Advances in computational imaging techniques have enabled the extraction of high-dimensional quantitative data from medical images, capturing tumor characteristics such as shape, texture, and intensity [17,18]. This approach, known as radiomics, provides an objective, robust, and reproducible framework for image interpretation and facilitates the identification of imaging biomarkers that may reflect intratumoral heterogeneity beyond the limits of visual assessment [19]. Radiomics-based models have demonstrated promising performance across multiple domains. Consequently, radiomics has attracted increasing interest in head and neck oncology—particularly in laryngeal cancer—given its potential to enhance staging accuracy and improve prognostic stratification [20].

Despite growing enthusiasm, evidence regarding the prognostic value of radiomic features in T3 LC remains limited, and findings have been inconsistent due to heterogeneous cohorts and methodological variability. Moreover, the existing literature has predominantly focused on pre-treatment radiomics for prognosis prediction or evaluation of anatomical tumor extension, whereas post-treatment imaging—where distinguishing radiation-related changes from residual or recurrent disease is particularly challenging—has received comparatively little attention. Therefore, the present study aimed to develop and validate a radiomics-derived combined model, integrating contrast-enhanced post-treatment CT features with clinical parameters, to predict local progression in patients with T3-stage laryngeal cancer treated with definitive organ preservation therapy.

## 2. Materials and Methods

### 2.1. Ethical Consideration

This single-center, retrospective cohort study was conducted on patients with locally advanced laryngeal cancer who presented to the Department of Otolaryngology–Head and Neck Surgery at Çukurova University between 2012 and 2023. The study protocol was in accordance with the Declaration of Helsinki, and ethical approval was granted by the Çukurova University Faculty of Medicine Non-Interventional Clinical Research Ethics Committee (meeting no: 145, decision no: 51).

### 2.2. Sample Size Determination and Patient Cohort

**Figure 1:**
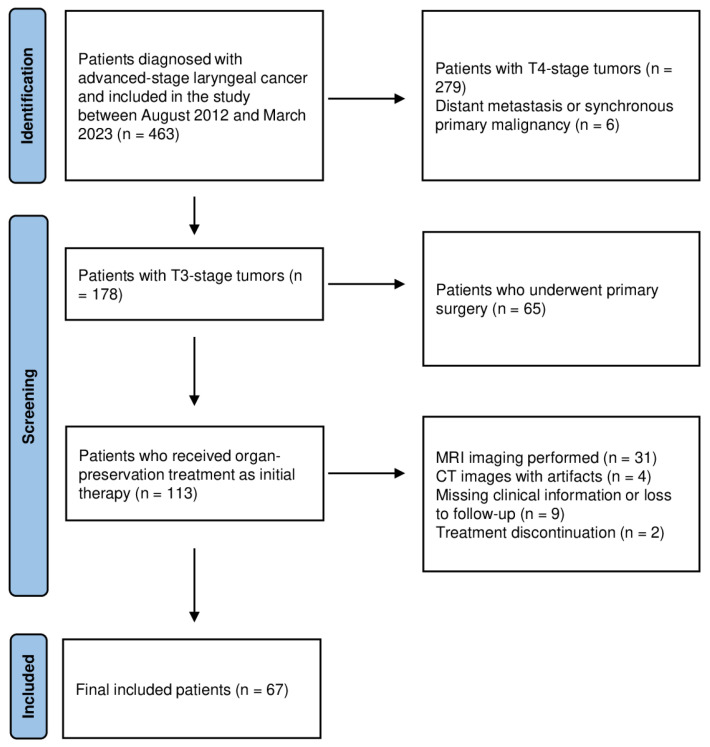
Patient selection flowchart showing inclusion and exclusion criteria of the study cohort.

In the sample size calculation, assuming a type I error rate (α) of 0.05, a statistical power of 80%, and an effect size of d = 0.2, the minimum required sample size was calculated as 67. A total of 463 patients applied during the study period, and 67 of them were included in the study according to the inclusion and exclusion criteria (Figure 1).

All patients underwent a standard otolaryngological evaluation, including direct laryngoscopy and tissue biopsy for subsequent histopathological assessment. In addition, radiological imaging modalities were utilized to improve diagnostic accuracy. Clinical and demographic variables were retrospectively collected from electronic medical records and standardized pretreatment clinical documentation. The following parameters were documented for each patient: age at diagnosis, sex, smoking status, alcohol use, primary tumor location, multiregional invasion, vocal cord fixation, and radiological suspicion of cartilage invasion.

Multiregional invasion was defined as tumor extension involving more than one laryngeal region/subsite (e.g., paraglottic, pre-epiglottic, post-cricoid, subglottic region) on endoscopic and/or imaging assessment. Vocal cord fixation was recorded based on pretreatment laryngoscopic examination findings. Suspicion of cartilage invasion was defined according to pretreatment imaging (contrast-enhanced CT and/or MRI when available), based on radiologist interpretation and/or multidisciplinary review, including findings such as cartilage sclerosis, erosion, lysis, or abnormal enhancement suggestive of invasion. Tumor staging was determined according to the 8th edition of the AJCC-TNM staging system [13]. Patients were included in the study if they met the following criteria:

1. Newly diagnosed, pathologically confirmed T3-stage laryngeal squamous cell carcinoma without evidence of distant metastasis.
2. Treatment with a non-surgical organ-preservation protocol.
3. Availability of contrast-enhanced CT imaging performed both prior to treatment and 3 months after completion of therapy.
4. Receipt of concurrent CRT, with cisplatin as the sole concurrent chemotherapy agent (cumulative dose 200 mg/m^2^). The radiotherapy dose, expressed as the equivalent dose in 2-Gy fractions (EQD2), was ≥66.0 Gy, delivered to the primary laryngeal site within 70 days. Patients who received induction chemotherapy were excluded.
5. No evidence of synchronous primary malignancies.

Following treatment, patients were subject to regular follow-up visits, with initial appointments occurring every 2–3 months for the first year, every 4–6 months for the subsequent 2–5 years, and annually after the fifth year. In the case of suspected recurrence, direct laryngoscopy and subsequent biopsy were performed. Local progression was recorded only when histopathology confirmed a viable tumor on direct laryngoscopy–guided biopsy. Although post-treatment edema and fibrosis occasionally delayed clinical suspicion, all cases categorized as local progression were ultimately biopsy-proven. The patients were observed until death or the final follow-up in July 2025.

The primary endpoint was local progression-free survival (LPFS), defined as the interval from the end of treatment to biopsy-proven residual or recurrent primary tumor; patients without an event were censored at the last disease assessment. The analysis focused on the primary laryngeal tumor; neck disease was not segmented for radiomics and was not included in the endpoint definition.

### 2.3. Treatment Protocol

All patients were treated with concurrent CRT, with cisplatin as the sole concurrent chemotherapy agent (cumulative dose: 200 mg/m^2^). The radiotherapy dose delivered to the primary laryngeal site, expressed as the EQD2, was ≥66.0 Gy, and treatment was completed within 70 days.

### 2.4. Imaging and Radiomics Protocol

Imaging data were acquired using a 160-multislice Toshiba Aquilion (Otawara, Japan) CT scanner. Iohexol-containing Omnipaque (GE Healthcare, Chicago, IL, USA) was used as the contrast agent. Contrast-enhanced neck CT scans were performed with patients in the supine position, and images were obtained with a display field of view of 51–60 cm (transverse) × 36–40 cm (antero-posterior), a tube voltage of 120–135 kV, and a tube current of 40–170 mA. The images were acquired using slice thickness protocols of 3 mm/1.5 mm spacing and 5 mm spacing and were evaluated in axial, coronal, and sagittal planes with slice thicknesses of 3–5 mm, covering the region from the base of the tongue to the trachea.

**Figure 2:**
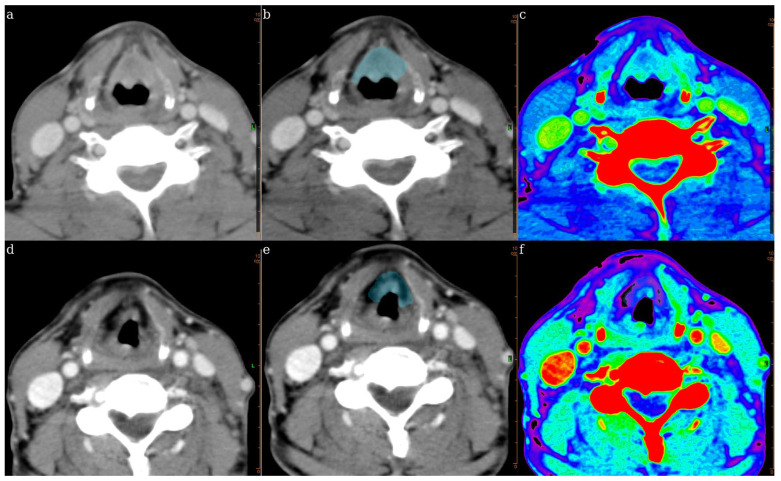
Representative axial CT images of a T3 laryngeal cancer patient before and after radiotherapy. Top row (**a**–**c**): Pre-treatment images showing (**a**) original CT, (**b**) tumor segmentation, and (**c**) texture-mapped color scale. Bottom row (**d**–**f**): Post-treatment images showing (**d**) original CT, (**e**) updated segmentation, and (**f**) post-treatment texture-mapped color scale. L, left side.

Two radiologists (5 and 14 years’ experience) reviewed all cases, blinded to clinical outcomes. Post-treatment primary tumor regions of interest (ROI) were manually segmented in 3D by consensus on the resampled images. Per our institutional surveillance protocol, post-treatment contrast-enhanced CT was performed routinely at ~3 months after completion of CRT for response assessment. The CT scans used for radiomics analysis were obtained at this standardized 3-month time point (or very close to it), minimizing timing-related variability. Pre-treatment scans were used only for anatomical correspondence (e.g., to localize the treated primary) and were not used for radiomic feature extraction or modeling. Radiomic features (shape, first-order histogram, and texture families including GLDM, GLCM, GLRLM, GLSZM) were computed using OLEA Sphere 3.0-SP28 software (Olea Medical^®^, La Ciotat, France), yielding 111 features per case (Figure 2).

### 2.5. Radiomics Score

Among 111 extracted radiomic features, candidate variables representing distinct tumor phenotypes were evaluated according to the PyRadiomics feature classes [21]: (i) shape features reflecting tumor morphology, (ii) first-order statistics derived from voxel intensity distributions, and (iii) texture features quantifying spatial relationships and intratumoral heterogeneity. The discriminative performance of radiomic features for local progression was assessed using receiver operating characteristic (ROC) analysis, and features with AUC > 0.60 were considered for inclusion. The six features with the highest AUC values were selected to construct the radiomics risk score and were distributed across PyRadiomics classes as follows: one shape feature (Original Shape Maximum 2D Diameter Row), two first-order features (Original First Order 10th Percentile, Original First Order Median), and three texture features including GLCM (Original GLCM Correlation), NGTDM (Original NGTDM Strength), and GLDM (Original GLDM Dependence Non-Uniformity) (Table 1). Optimal cutoff values were determined using Youden’s J index. For five features (Maximum 2D Diameter Row, 10th Percentile, Median, GLCM Correlation, and GLDM Dependence Non-Uniformity), values above the cutoff were scored as 1 (high-risk) and values below as 0 (low-risk), whereas for NGTDM Strength, values below the cutoff were scored as 1 and values above as 0. The radiomics score was calculated as the sum of these binary points, yielding a total score ranging from 0 to 6, with higher values indicating an increased risk of local progression.

### 2.6. Statistical Analysis

Statistical analyses were performed using JAMOVI (v2.6.44). Normality was assessed with the Shapiro–Wilk test, and parametric or non-parametric tests were applied accordingly. Normally distributed continuous variables were reported as mean ± standard deviation and compared using the independent samples *t*-test. Group comparisons were performed using the *t*-test and Fisher’s exact test, as appropriate. Time-to-event outcomes were analyzed using Kaplan–Meier curves and Cox proportional hazard regression. ROC analysis was used to evaluate discriminative performance, and feature AUCs were compared using DeLong’s test. A two-sided *p* < 0.05 was considered statistically significant.

## 3. Results

The mean age of the patients included in the study was 59.6 ± 9.42 years (range: 38–80). During follow-up, 37.3% of patients developed local progression. When sociodemographic and clinical characteristics were compared according to local progression status, patients who experienced local progression had a significantly lower mean age and a higher rate of cartilage invasion. Although all patients in the local progression group were male, no statistically significant difference in sex distribution was observed between the groups. Moreover, alcohol consumption, smoking status, primary tumor localization, multiregional invasion, and vocal cord fixation did not differ significantly between patients with and without local progression (Table 2).

**Figure 3:**
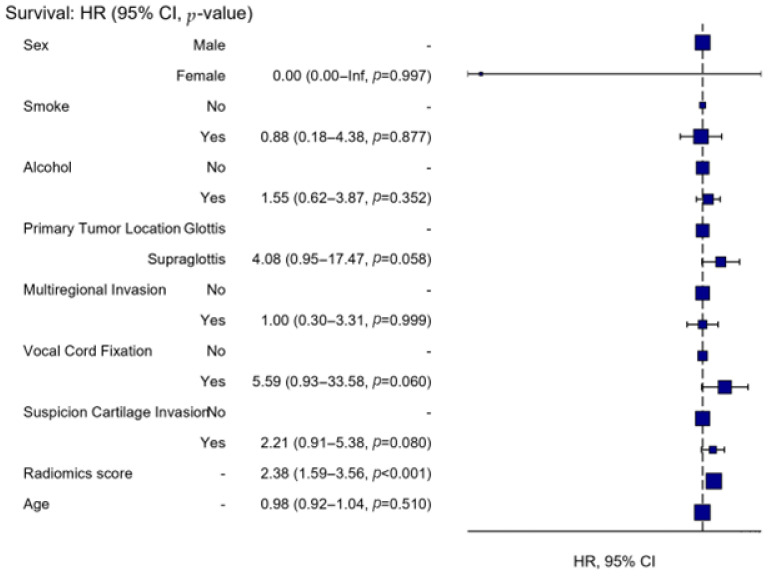
Forest plot of the multivariable Cox proportional hazards model for local progression. Hazard ratios (HRs) with 95% confidence intervals (CIs) are shown for the included clinical covariates and the radiomics score. Squares indicate HR point estimates and horizontal lines represent 95% CIs; the dashed vertical line denotes HR = 1.0. *p*-values are provided for each covariate.

A Cox proportional hazard regression model developed to predict the risk of local progression in patients with T3 laryngeal cancer was statistically significant (*p* < 0.001) and demonstrated high discriminative ability (concordance index = 0.855, SE = 0.030). The independent variables included in the model explained 51% of the variance (R^2^ = 0.514), indicating good overall model performance. After adjustment for covariates, the radiomics score remained an independent and strong predictor of local progression. Each one-unit increase in the radiomics score was associated with a 2.38-fold increase in the risk of local progression (HR = 2.38, 95% CI: 1.59–3.56, *p* < 0.001). In the multivariable model, cartilage invasion, supraglottic tumor localization, vocal cord fixation, smoking, alcohol use, multiregional invasion, and age were not independently associated with local progression (Table 3, Figure 3).

Kaplan–Meier analysis performed to estimate LPFS across the consolidated radiomics score groups was statistically significant (*p* < 0.001), demonstrating substantial differences in time-to-event outcomes. The mean time to local progression was 41.7 months for patients with a high radiomics score (>3), compared to 122.6 months for those with a low radiomics score (≤3). Univariable Cox regression analysis indicated that patients in the low radiomics score group had an 88% lower risk of local progression compared to the high-score group (HR = 0.12; 95% CI: 0.04–0.35; *p* < 0.001) (Table 4). Although the theoretical range of the radiomics score is 0–6, no patient in the dataset achieved a score of 6; thus, this category was omitted from the analysis.

LPFS rates differed significantly across the consolidated radiomics score strata (Table 5). In patients with a high radiomics score (>3), the 1-year LPFS rate was 62.8%, which declined to 29.4% at 3 years and 23.5% at 5 years. In contrast, patients with a lower radiomics score (≤3) demonstrated more robust survival outcomes, with a 1-year LPFS rate of 90.9% and stable 3- and 5-year rates of 87.9%. Although the theoretical range of the radiomics score is 0–6, no patient in our dataset achieved a score of 6; therefore, this category was not included in the survival analysis. Detailed LPFS rates at 1, 3, and 5 years are provided in Table 5.

**Figure 4:**
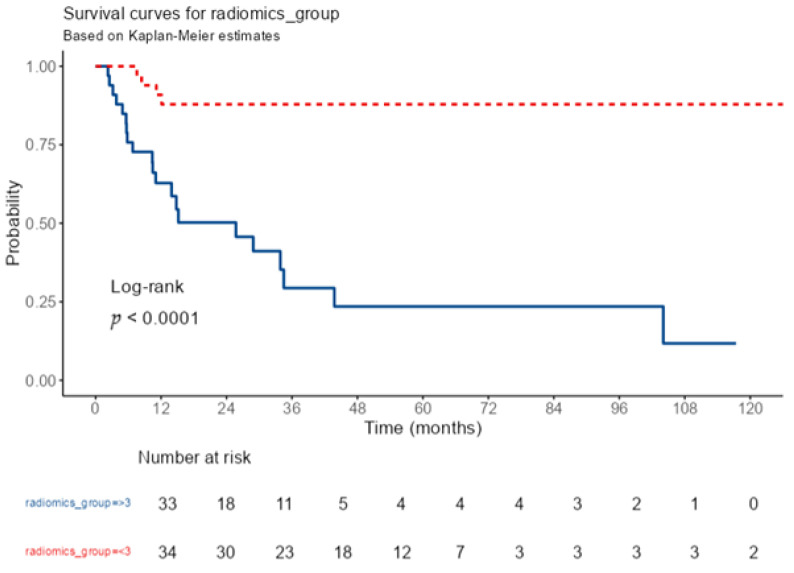
Kaplan–Meier local progression-free survival analysis stratified by radiomics group. The solid blue line indicates patients with radiomics group ≥ 3, whereas the dashed red line indicates patients with radiomics group < 3. Survival distributions were compared using the log-rank test (*p* < 0.0001).

The total follow-up time was 2290.7 patient-months, during which 25 events of local progression occurred. The overall incidence rate was 1.09 events per 100 patient-months (95% CI: 0.71–1.61). The LPFS curves stratified by radiomics score are shown in Figure 4.

## 4. Discussion

Prognosis and treatment response in T3-stage laryngeal cancer may vary considerably, posing substantial challenges for clinicians during evaluation and decision-making. Although a paradigm shift toward organ-preservation strategies [6,7] has occurred following landmark clinical trials, the role of organ-preserving therapies in the management of T3 LC remains controversial. The T3 category represents a biologically heterogeneous group of tumors, encompassing a broad spectrum of disease—from limited glottic involvement with mild vocal cord fixation to more infiltrative tumors extending into the paraglottic space. While surgeons can relatively anticipate outcomes of surgical management based on the extent and adequacy of resection, predictions regarding non-surgical treatment outcomes remain more uncertain. The increasing adoption of organ-preserving approaches and the inherent unpredictability of treatment results have introduced specific challenges, including concerns regarding the reliability of prognostic assessment and the timely detection of recurrent or residual disease.

Assessment strategies after CRT in patients with T3 LC are of critical importance due to the complex anatomy of the larynx, treatment-related morphological alterations such as edema and fibrosis, and the pronounced intratumoral heterogeneity [22]. Until recently, the interpretation of imaging modalities largely relied on subjective visual assessment by the human observer. However, with the advent of artificial intelligence, advanced computational algorithms have enabled the non-invasive, reproducible, and high-throughput extraction of quantitative information—such as shape, volume, voxel intensity, and texture—from conventional medical images [23]. These features are often imperceptible to the human eye and provide valuable insights into the tumor microenvironment, thereby complementing clinical evaluation.

Radiomics-based image texture analysis has been explored in LC for tumor characterization, staging, anatomical extension, risk stratification, and prognostication [24]. Most studies have focused on predicting clinical outcomes—such as prognosis, local control, and survival—using pretreatment imaging techniques. Bogowicz et al. [25] demonstrated that a radiomic signature comprising three CT-based features significantly predicted local control in 149 HNC patients (C-index: 0.78). In another study [26], a combined model integrating both clinical and radiomic features was reported to demonstrate the highest performance for predicting progression-free survival (C-index = 0.808). Similarly, Rajgor et al. [27] showed that the inclusion of radiomic features improved the model’s discrimination, yielding a higher C-index (0.759) compared with the model based solely on clinicopathological variables (0.655). Zhai et al. [28] reported that CT-based imaging biomarkers performed similarly or slightly better than clinical variables in predicting treatment outcomes in a cohort of 444 HNC patients. Most prior studies were conducted on heterogeneous HNC cohorts without subsite distinction [25,26,27,28,29]. While studies highlighted the prognostic value of radiomics, their applicability to specific subsites remains limited. In this context, we analyzed a homogeneous cohort consisting exclusively of T3 LC patients. This targeted approach provides more clinically relevant prognostic insights for a group characterized by treatment uncertainty.

As highlighted above, the majority of radiomics studies in head and neck oncology have predominantly relied on pretreatment imaging. Nevertheless, post-treatment CT—capturing tissue alterations within the anatomical region corresponding to the pretreatment ROI—may provide complementary information regarding residual tumor biology, treatment-induced microstructural changes, and response dynamics. Accordingly, such post-therapy imaging biomarkers could be clinically relevant for refining risk stratification in terms of treatment response, local control, and long-term survival outcomes. Despite this rationale, the prognostic value of post-treatment imaging for predicting local control and survival remains insufficiently explored. Lin et al. [30] reported that radiomic models derived from mid-radiotherapy imaging, both peritumoral (AUC = 0.77) and intratumoral (AUC = 0.79), outperformed pretreatment models (AUC = 0.62) and served as independent predictors of both overall survival and progression-free survival. However, this analysis included laryngeal and hypopharyngeal carcinomas without stage-specific stratification. Similarly, Rana et al. [31], in a small cohort of 24 patients, suggested that CT perfusion metrics may be informative for evaluating CRT response. Yet the potential of perfusion-derived parameters to predict post-treatment disease-free survival was not assessed. Moreover, the inclusion of heterogeneous primary HNC subsites without subsite-level analyses further limits the generalizability of these findings.

Post-treatment assessment after definitive CRT in T3 laryngeal cancer typically relies on clinical examination and laryngoscopy, with imaging used to support response evaluation and to guide direct laryngoscopy with biopsy when recurrence is suspected. However, in the early post-treatment period, mucosal edema, inflammation, and fibrosis can mimic or mask residual/recurrent disease and may render conventional anatomic imaging (CT/MRI) equivocal, thereby delaying definitive tissue confirmation [32,33]. Moreover, even advanced functional imaging may be affected by treatment-related inflammatory changes; for example, FDG-PET is commonly recommended at ≥12 weeks to reduce false positives, yet false-positive uptake can still occur due to post-radiation mucositis, necrosis, and reactive changes [34,35]. In this context, quantitative CT-derived radiomics may complement standard surveillance by capturing subvisual patterns of tissue heterogeneity not appreciable on routine visual assessment, potentially providing an objective risk signal that could prompt earlier endoscopic reassessment and timely biopsy in patients at higher risk of biopsy-confirmed local progression.

Collectively, these limitations underscore a critical evidence gap regarding the prognostic utility of post-treatment CT-derived radiomic signatures in LC, particularly within homogeneous, stage-defined cohorts treated with organ-preservation protocols. Addressing this gap may enable more refined risk stratification beyond conventional clinical variables and facilitate earlier identification of patients at high risk of local progression.

Therefore, in the present study, we developed a combined prognostic model incorporating clinical biomarkers and a radiomics score derived from six selected radiomic features to predict local progression risk. The integrated model demonstrated high discriminative performance (C-index = 0.855). Notably, the radiomics score remained an independent and robust predictor of local progression after adjustment for other covariates, with each one-unit increase in the radiomics score associated with a 2.38-fold higher risk of local progression (HR = 2.38, 95% CI: 1.59–3.56, *p* < 0.001). Furthermore, compared to patients with a radiomic score greater than 3, the risk of local progression was 88% lower in patients with a score of 3 or lower (HR = 0.12). From a clinical perspective, a post-treatment CT-derived radiomics risk score could potentially support risk-adapted surveillance after CRT in T3 laryngeal cancer. In routine practice, post-CRT edema and fibrosis may delay clinical suspicion and biopsy confirmation; therefore, an objective imaging-based risk signal may help identify patients who could benefit from closer follow-up, earlier endoscopic reassessment, and a lower threshold for direct laryngoscopy with biopsy when findings are equivocal. Conversely, patients classified as at lower risk might reasonably continue with standard surveillance intervals. Importantly, these proposed applications are hypothesis-generating; prospective evaluation and external validation are required to determine clinical utility, optimal thresholds, and whether score-guided surveillance improves patient outcomes.

This study has several limitations. First, the single-center, retrospective design and modest sample size may introduce selection bias. Additionally, the high number of initial radiomics features screened (n = 111) relative to the number of local progression events (n = 25) introduces a potential risk of selection instability and optimistic performance estimates. Although we have reported 95% confidence intervals for our AUC estimates to reflect this uncertainty, the risk of multiplicity cannot be entirely eliminated; therefore, our findings should be considered exploratory, and the developed 6-feature score requires further validation in larger, independent, multi-center cohorts. Second, we intentionally focused on post-treatment contrast-enhanced CT due to its routine clinical availability; however, MRI and/or FDG-PET were not incorporated and may provide complementary prognostic information in a multimodal framework. Third, radiomic analysis was restricted to the primary tumor; nodal disease was not segmented, and the study was primarily designed around LPFS rather than overall survival, limiting conclusions regarding nodal-driven outcomes and long-term endpoints. Fourth, radiomic features may vary across software implementations, acquisition protocols, scanners, and the timing of post-treatment imaging; although a consistent workflow was applied, broader standardization and harmonization strategies may further improve generalizability. Body mass index and nutritional/body composition parameters were not consistently available in this retrospective cohort and should be incorporated in future prospective studies.

## 5. Conclusions

Local progression after organ-preserving therapy in T3 laryngeal cancer remains difficult to anticipate using conventional assessment alone. In this retrospective single-center study, integrating a post-treatment CT-derived radiomics score with clinical parameters yielded a combined model with favorable performance for risk stratification. While these findings are preliminary and warrant external validation, this approach may support more personalized follow-up strategies by complementing routine clinical evaluation.

## Figures and Tables

**Table 1:** ROC-Derived Performance Metrics of the Six Radiomic Features Included in the Radiomics Score for Predicting Local Progression.

	AUC (95% CI)	*p*	Optimal Cut-Off	Sensitivity	Specificity
Original Shape Maximum 2D Diameter Row	0.700 (0.575–0.825)	0.002	≥30.04	84.00	54.76
Original First Order 10th Percentile	0.729 (0.606–0.851)	<0.001	≥1.19	92.00	52.38
Original First Order Median	0.729 (0.606–0.851)	<0.001	≥1.23	92.00	50.00
Original Gray Level Co-occurrence Matrix Correlation	0.680 (0.552–0.808)	0.006	≥0.41	92.00	50.00
Original Neighboring Gray Tone Difference Matrix Strength	0.636 (0.501–0.772)	0.049	≤9.85	92.00	42.86
Original Gray Level Dependence Matrix Dependence Non-Uniformity	0.650 (0.509–0.790)	0.037	≥315.61	76.00	57.14

ROC, receiver operating characteristic; AUC, area under the curve. Optimal cut-off values were determined using the Youden index. *p* values indicate the statistical significance of AUC values compared with 0.5 (no discrimination). Sensitivity and specificity are reported for predicting local progression.

**Table 2:** Baseline Clinical and Demographic Characteristics Stratified by Local Progression Status.

		Local Progression Mean ± S.D. or n (%)		
Variables		No (n: 42)	Yes (n: 25)	*p*
Age		61.4 ± 9.3	56.6 ± 9.1	0.045
Sex	Male	36 (85.7)	25 (100.0)	0.124
	Female	6 (14.3)	0 (0.0)	
Smoking Status	No	7 (16.7)	2 (8.0)	0.525
	Yes	35 (83.3)	23 (92.0)	
Alcohol Use	No	29 (69.0)	13 (52.0)	0.257
	Yes	13 (31.0)	12 (48.0)	
Primary Tumor Subsite	Glottis	29 (69.0)	14 (56.0)	0.416
	Supraglottis	13 (31.0)	11 (44.0)	
Multiregional Invasion	No	34 (81.0)	16 (64.0)	0.211
	Yes	8 (19.0)	9 (36.0)	
Vocal Cord Fixation	No	14 (33.3)	8 (32.0)	1.000
	Yes	28 (66.7)	17 (68.0)	
Suspicion of Cartilage Invasion	No	38 (90.5)	17 (68.0)	0.046
	Yes	4 (9.5)	8 (32.0)	

S.D., standard deviation. Values are presented as mean ± S.D. or n (%). Continuous variables were compared using the independent samples *t*-test, and categorical variables using the Chi-square or Fisher’s exact test, as appropriate. Significant values are indicated in bold (*p* < 0.05).

**Table 3:** Univariable and Multivariable Cox Proportional Hazard Regression Analyses for Local Progression.

Independent Variables		All	HR (C.I.) (Univariable)	HR (C.I.) (Multivariable)
Sex	Male	61 (91.0)	-	-
	Female	6 (9.0)	0.00 (0.00-Inf, *p* = 0.998)	0.00 (0.00-Inf, *p* = 0.997)
Smoking Status	No	9 (13.4)	-	-
	Yes	58 (86.6)	1.99 (0.47–8.47, *p* = 0.351)	0.88 (0.18–4.38, *p* = 0.877)
Alcohol Use	No	42 (62.7)	-	-
	Yes	25 (37.3)	1.95 (0.89–4.29, *p* = 0.096)	1.55 (0.62–3.87, *p* = 0.352)
Primary Tumor Subsite	Glottis	43 (64.2)	-	-
	Supraglottis	24 (35.8)	1.84 (0.83–4.10, *p* = 0.134)	4.08(0.95–17.47, *p* = 0.058)
Multiregional Invasion	No	50 (74.6)	-	-
	Yes	17 (25.4)	2.16 (0.93–4.98, *p* = 0.072)	1.00 (0.30–3.31, *p* = 0.999)
Vocal Cord Fixation	No	22 (32.8)	-	-
	Yes	45 (67.2)	0.83 (0.36–1.94, *p* = 0.666)	5.59 (0.93–33.58, *p* = 0.060)
Suspicion of Cartilage Invasion	No	55 (82.1)	-	-
	Yes	12 (17.9)	2.94 (1.25–6.93, *p* = 0.014)	2.21 (0.91–5.38, *p* = 0.080)
Radiomics Score	Mean (SD)	3.8 (1.9)	2.29 (1.60–3.29, *p* < 0.001)	2.38 (1.59–3.56, *p* < 0.001)
Age	Mean (SD)	59.6 (9.4)	0.95 (0.91–1.00, *p* = 0.050)	0.98 (0.92–1.04, *p* = 0.510)

HR, hazard ratio; CI, confidence interval; SD, standard deviation. Values are presented as n (%) or mean (SD), as appropriate. HRs are reported with 95% confidence intervals. Univariable and multivariable Cox proportional hazard regression models were used to evaluate predictors of local progression. *p* values < 0.05 were considered statistically significant and are shown in bold.

**Table 4:** Local Progression-Free Survival According to Radiomics Score Categories: Event Rates and Univariable Cox Regression.

Radiomics Score	Records	Local Progression (n)	Mean (Month)	S.D.	All	HR (C.I.) (Univariable)
>3	33	21	41.7	9.99	33 (49.3)	-
≤3	34	4	122.6	7.29	34 (50.7)	0.12 (0.04–0.35, *p* < 0.001)

HR, hazard ratio; CI, confidence interval; S.D., standard deviation. Note: The radiomics score was dichotomized into two risk groups as low-risk (≤3) and high-risk (>3) to ensure more balanced sample sizes across strata and to enhance the statistical robustness of the survival estimates.

**Table 5:** 1-, 3-, and 5-Year Local Progression-Free Survival Rates by Radiomics Score Category.

					95% C.I.	
Radiomics Score	Time (Year)	Number at Risk	Number of Events	LPFS (%)	Lower	Upper
>3	1	18	12	62.8	48.1	82.0
>3	3	5	7	29.4	15.2	56.7
>3	5	4	1	23.5	10.7	51.8
≤3	1	30	3	90.9	81.6	100.0
≤3	3	18	1	87.9	77.4	99.8
≤3	5	7	0	87.9	77.4	99.8

LPFS, local progression-free survival; CI, confidence interval. LPFS rates were estimated using the Kaplan–Meier method. “Number at risk” indicates patients remaining under follow-up without local progression at each time point.

## Data Availability

The datasets generated and/or analyzed data during the current study are available from the corresponding author upon reasonable request.

## Abbreviations

The following abbreviations are used in this manuscript:

LC	Laryngeal cancer
LPFS	Local progression-free survival
AJCC	American Joint Committee on Cancer
HNC	Head and neck cancer
CRT	Chemoradiotherapy
EQD2	Equivalent dose in 2-Gy fractions
ROI	Region of interest
ROC	Receiver operating characteristic
AUC	Area under curve
GLCM	Gray level co-occurrence matrix
GLDM	Gray level dependence matrix
NGTDM	Neighboring gray tone difference matrix
HR	Hazard ratio
CI	Confidence interval
SD	Standard deviation

## References

[1] Steuer C.E., El-Deiry M., Parks J.R., Higgins K.A., Saba N.F. (2017). An update on larynx cancer. CA Cancer J. Clin..

[2] International Agency for Research on Cancer (IARC) (2022). Cancer Today—Larynx Fact Sheet (GLOBOCAN 2022).

[3] Bray F., Laversanne M., Sung H., Ferlay J., Siegel R.L., Soerjomataram I., Jemal A. (2024). Global cancer statistics 2022: GLOBOCAN estimates of incidence and mortality worldwide for 36 cancers in 185 countries. CA Cancer J. Clin..

[4] Forastiere A.A., Ismaila N., Wolf G.T. (2018). Use of Larynx-Preservation Strategies in the Treatment of Laryngeal Cancer: American Society of Clinical Oncology Clinical Practice Guideline Update Summary. J. Oncol. Pract..

[5] Ravanelli M., Rondi P., Di Meo N., Farina D. (2024). The added value of radiomics in determining patient responsiveness to laryngeal preservation strategies. Curr. Opin. Otolaryngol. Head. Neck Surg..

[6] Wolf G.T., Fisher S.G., Hong W.K., Hillman R., Spaulding M., Laramore G.E., Endicott J.W., McClatchey K., Henderson W.G., Department of Veterans Affairs Laryngeal Cancer Study Group (1991). Induction chemotherapy plus radiation compared with surgery plus radiation in patients with advanced laryngeal cancer. N. Engl. J. Med..

[7] Forastiere A.A., Goepfert H., Maor M., Pajak T.F., Weber R., Morrison W., Glisson B., Trotti A., Ridge J.A., Chao C. (2003). Concurrent chemotherapy and radiotherapy for organ preservation in advanced laryngeal cancer. N. Engl. J. Med..

[8] Hoffman H.T., Porter K., Karnell L.H., Cooper J.S., Weber R.S., Langer C.J., Ang K., Gay G., Stewart A., Robinson R.A. (2006). Laryngeal cancer in the United States: Changes in demographics, patterns of care, and survival. Laryngoscope.

[9] Prades J.M., Lallemant B., Garrel R., Reyt E., Righini C., Schmitt T., Remini N., Saban-Roche L., Timoshenko A.P. (2010). Randomized phase III trial comparing induction chemotherapy followed by radiotherapy to concomitant chemoradiotherapy for laryngeal preservation in T3M0 pyriform sinus carcinoma. Acta Otolaryngol..

[10] Lee N.Y., O’Meara W., Chan K., Della-Bianca C., Mechalakos J.G., Zhung J., Wolden S.L., Narayana A., Kraus D., Shah J.P. (2007). Concurrent chemotherapy and intensity-modulated radiotherapy for locoregionally advanced laryngeal and hypopharyngeal cancers. Int. J. Radiat. Oncol. Biol. Phys..

[11] Juloori A., Koyfman S.A., Geiger J.L., Joshi N.P., Woody N.M., Burkey B.B., Scharpf J., Lamarre E.L., Prendes B., Adelstein D.J. (2018). Definitive Chemoradiation in Locally Advanced Squamous Cell Carcinoma of the Hypopharynx: Long-term Outcomes and Toxicity. Anticancer Res..

[12] Megwalu U.C., Sikora A.G. (2014). Survival outcomes in advanced laryngeal cancer. JAMA Otolaryngol. Head Neck Surg..

[13] Amin M.B., Greene F.L., Edge S.B., Compton C.C., Gershenwald J.E., Brookland R.K., Meyer L., Gress D.M., Byrd D.R., Winchester D.P. (2017). The Eighth Edition AJCC Cancer Staging Manual: Continuing to build a bridge from a population-based to a more “personalized” approach to cancer staging. CA Cancer J. Clin..

[14] Dziegielewski P.T., O’Connell D.A., Klein M., Fung C., Singh P., Mlynarek M.A., Fung D., Harris J.R., Seikaly H. (2012). Primary total laryngectomy versus organ preservation for T3/T4a laryngeal cancer: A population-based analysis of survival. J. Otolaryngol. Head Neck Surg..

[15] Broggi G., Maniaci A., Lentini M., Palicelli A., Zanelli M., Zizzo M., Koufopoulos N., Salzano S., Mazzucchelli M., Caltabiano R. (2024). Artificial Intelligence in Head and Neck Cancer Diagnosis: A Comprehensive Review with Emphasis on Radiomics, Histopathological, and Molecular Applications. Cancers.

[16] Glastonbury C.M., Parker E.E., Hoang J.K. (2010). The postradiation neck: Evaluating response to treatment and recognizing complications. AJR Am. J. Roentgenol..

[17] Mortensen L.S., Johansen J., Kallehauge J., Primdahl H., Busk M., Lassen P., Alsner J., Sørensen B.S., Toustrup K., Jakobsen S. (2012). FAZA PET/CT hypoxia imaging in patients with squamous cell carcinoma of the head and neck treated with radiotherapy: Results from the DAHANCA 24 trial. Radiother. Oncol..

[18] Bahig H., Lapointe A., Bedwani S., de Guise J., Lambert L., Filion E., Roberge D., Létourneau-Guillon L., Blais D., Ng S.P. (2019). Dual-energy computed tomography for prediction of loco-regional recurrence after radiotherapy in larynx and hypopharynx squamous cell carcinoma. Eur. J. Radiol..

[19] van Timmeren J.E., Cester D., Tanadini-Lang S., Alkadhi H., Baessler B. (2020). Radiomics in medical imaging-“how-to” guide and critical reflection. Insights Imaging.

[20] Chiesa-Estomba C.M., Echaniz O., Larruscain E., Gonzalez-Garcia J.A., Sistiaga-Suarez J.A., Graña M. (2019). Radiomics and Texture Analysis in Laryngeal Cancer. Looking for New Frontiers in Precision Medicine through Imaging Analysis. Cancers.

[21] van Griethuysen J.J.M., Fedorov A., Parmar C., Hosny A., Aucoin N., Narayan V., Beets-Tan R.G.H., Fillion-Robin J.-C., Pieper S., Aerts H.J.W.L. (2017). Computational Radiomics System to Decode the Radiographic Phenotype. Cancer Res..

[22] Camp R.L., Dolled-Filhart M., Rimm D.L. (2004). X-tile: A new bio-informatics tool for biomarker assessment and outcome-based cut-point optimization. Clin. Cancer Res..

[23] Forghani R., Chatterjee A., Reinhold C., Pérez-Lara A., Romero-Sanchez G., Ueno Y., Bayat M., Alexander J.W.M., Kadi L., Chankowsky J. (2019). Head and neck squamous cell carcinoma: Prediction of cervical lymph node metastasis by dual-energy CT texture analysis with machine learning. Eur. Radiol..

[24] Rajgor A.D., Patel S., McCulloch D., Obara B., Bacardit J., McQueen A., Aboagye E., Ali T., O’hara J., Hamilton D.W. (2021). The application of radiomics in laryngeal cancer. Br. J. Radiol..

[25] Bogowicz M., Riesterer O., Ikenberg K., Stieb S., Moch H., Studer G., Guckenberger M., Tanadini-Lang S. (2017). Computed Tomography Radiomics Predicts HPV Status and Local Tumor Control After Definitive Radiochemotherapy in Head and Neck Squamous Cell Carcinoma. Int. J. Radiat. Oncol. Biol. Phys..

[26] Nakajo M., Nagano H., Jinguji M., Kamimura Y., Masuda K., Takumi K., Tani A., Hirahara D., Kariya K., Yamashita M. (2023). The usefulness of machine-learning-based evaluation of clinical and pretreatment 18F-FDG-PET/CT radiomic features for predicting prognosis in patients with laryngeal cancer. Br. J. Radiol..

[27] Rajgor A.D., Kui C., McQueen A., Cowley J., Gillespie C., Mill A., Rushton S., Obara B., Bigirumurame T., Kallas K. (2024). Computed tomography-based radiomic markers are independent prognosticators of survival in advanced laryngeal cancer: A pilot study. J. Laryngol. Otol..

[28] Zhai T.T., Langendijk J.A., van Dijk L.V., Halmos G.B., Witjes M.J., Oosting S.F., Noordzij W., Sijtsema N.M., Steenbakkers R.J. (2019). The prognostic value of CT-based image-biomarkers for head and neck cancer patients treated with definitive (chemo-) radiation. Oral Oncol..

[29] Agarwal J.P., Sinha S., Goda J.S., Joshi K., Mhatre R., Kannan S., Laskar S.G., Gupta T., Murthy V., Budrukkar A. (2020). Tumor radiomic features complement clinico-radiological factors in predicting long-term local control and laryngectomy free survival in locally advanced laryngo-pharyngeal cancers. Br. J. Radiol..

[30] Lin C.H., Yan J.L., Yap W.K., Kang C.-J., Chang Y.-C., Tsai T.-Y., Chang K.-P., Liao C.-T., Hsu C.-L., Chou W.-C. (2023). Prognostic value of interim CT-based peritumoral and intratumoral radiomics in laryngeal and hypopharyngeal cancer patients undergoing definitive radiotherapy. Radiother. Oncol..

[31] Rana L., Sharma S., Sood S., Singh B., Gupta M.K., Minhas R., Jhobta A., Bhatia V., Venkat B. (2015). Volumetric CT perfusion assessment of treatment response in head and neck squamous cell carcinoma: Comparison of CT perfusion parameters before and after chemoradiation therapy. Eur. J. Radiol. Open.

[32] Agra I.M., Ferlito A., Takes R.P., Silver C.E., Olsen K.D., Stoeckli S.J., Strojan P., Rodrigo J.P., Filho J.G., Genden E.M. (2012). Diagnosis and treatment of recurrent laryngeal cancer following initial nonsurgical therapy. Head Neck.

[33] Hermans R. (2004). Post-treatment imaging of head and neck cancer. Cancer Imaging.

[34] Purohit B.S., Ailianou A., Dulguerov N., Becker C.D., Ratib O., Becker M. (2014). FDG-PET/CT pitfalls in oncological head and neck imaging. Insights Imaging.

[35] Dejanovic D., Specht L., Czyzewska D., Kiil Berthelsen A., Loft A. (2022). Response Evaluation Following Radiation Therapy With 18F-FDG PET/CT: Common Variants of Radiation-Induced Changes and Potential Pitfalls. Semin. Nucl. Med..

